# Functional screening of genes suppressing TRAIL-induced apoptosis: distinct inhibitory activities of Bcl-X_L_ and Bcl-2

**DOI:** 10.1038/sj.bjc.6600795

**Published:** 2003-03-18

**Authors:** I-K Kim, Y-K Jung, D-Y Noh, Y-S Song, C-H Choi, B-H Oh, E S Masuda, Y-K Jung

**Affiliations:** 1 Department of Life Science, Kwangju Institute of Science and Technology, Kwangju, Korea; 2 Department of Surgery, Seoul National University College of Medicine, Seoul, Korea; 3 Department of Pharmacology, College of Medicine, Chosun University, Kwangju, Korea; 4 Department of Life Science, Pohang University of Science and Technology, Pohang, Korea; 5 RIGEL Pharmaceutical Inc., South San Francisco, CA 94080, USA

**Keywords:** TRAIL, apoptosis, functional screening, Bcl-X_L_, caspase

## Abstract

TNF-related apoptosis-inducing ligand (TRAIL) is known to selectively induce apoptosis in various tumour cells. However, downstream-signalling of TRAIL-receptor is not well defined. A functional genetic screening was performed to isolate genes interfering with TRAIL-induced apoptosis using cDNA retroviral library. Bcl-X_L_ and FLIP were identified after DNA sequencing analysis of cDNA rescued from TRAIL-resistant clones. We found that increased expression of Bcl-X_L_, but not Bcl-2, suppressed TRAIL-induced apoptosis in tumour cells. Western blot and immunohistochemical analyses showed that expression of Bcl-X_L_, but not Bcl-2, was highly increased in human breast cancer tissues. Exposure of MDA-MB-231 breast tumour cells to TRAIL induced apoptosis accompanied by dissipation of mitochondrial membrane potential and enzymatic activation of caspase-3, -8, and -9. However, SK-BR-3 breast tumour cells exhibiting increased expression level of Bcl-X_L_ were resistant to TRAIL, though upon exposure to TRAIL, caspase-8 and Bid were activated. Forced expression of Bcl-X_L_, but not Bcl-2, desensitised TRAIL-sensitive MDA-MB-231 cells to TRAIL. Similar inhibitory effects were also observed in other tumour cells such as HeLa and Jurkat cells stably expressing Bcl-X_L_, but not Bcl-2. These results are indicative of the crucial and distinct function of Bcl-X_L_ and Bcl-2 in the modulation of TRAIL-induced apoptosis.

Tumour cells express several proteins that suppress apoptosis and thereby become resistant to various forms of therapy. Gene products controlling the balance between cell death and survival arise from an expanding family of genes, of which Bcl- 2 family is clearly associated with apoptosis inhibition (Chao and Korsmeyer, 1998). The antiapoptotic members of Bcl-2 gene family exert their antiapoptotic functions by preventing the release of cytochrome *c* from mitochondria to the cytosol and prevent the loss of mitochondrial outer membrane integrity by blocking both membrane hyperpolarisation and mitochondrial swelling (Vander-Heiden *et al*, 1997; Harris and Thompson, 2000).

Agents that induce apoptosis in cancer cells have recently attracted great attention. The apoptotic process can be triggered by pleiotropic ways, including activation of tumour necrosis factor receptor (TNF-R) family, *γ*-irradiation, and various chemotherapeutic agents, etc. The known signalling pathways induced by various apoptotic stimuli converge into a common death pathway either at mitochondrial step or finally at a step at which caspases are activated (Green and Reed, 1998; Thornberry and Lazebnik, 1998). TNF-related apoptosis-inducing ligand (TRAIL) is a member of the TNF family that is capable of inducing apoptosis in tumour cells examined (Wiley *et al*, 1995; Pitti *et al*, 1996). In animal model, TRAIL efficiently suppressed tumours with no detectable toxicity, suggesting that it could potentially serve as an useful chemotherapeutic agent (Ashkenazi *et al*, 1999; Walczak *et al*, 1999). While some studies raised questions of whether normal cell types were truly protected from TRAIL (Jo *et al*, 2000; Leverkus *et al*, 2000), the TRAIL currently being developed for clinical trials does not evoke these cytotoxic effects on hepatocytes (Lawrence *et al*, 2001; Qin *et al*, 2001).

TNF-related apoptosis-inducing ligand is highly homologous to FasL and TNF ligand family. Unlike other members, TRAIL is constitutively expressed in most tissues and cells (Wiley *et al*, 1995). TNF-related apoptosis-inducing ligand-R1 (DR4), TRAIL-R2 (DR5), TRAIL-R3 (DcR1), and TRAIL-R4 (DcR2) have been identified as TRAIL receptors (Pan *et al*, 1997a,1997b; Sheridan *et al*, 1997; Wu *et al*, 1997; Mongkolsapaya *et al*, 1998). While TRAIL-R1 and TRAIL-R2 contain a cytoplasmic death domain, TRAIL-R3 and TRAIL-R4 lack the death domain and bind to TRAIL without activation of apoptotic machinery. Though caspase-8 was recently reported to play a critical role in TRAIL-mediated apoptosis (Kim *et al*, 2000), the sequence of events occurring downstream of the receptors is not well understood. In the present study, we isolated Bcl-X_L_ as an inhibitory gene of TRAIL-induced apoptosis from cDNA library by expression screening assay. We found differential expression pattern of Bcl-2 and Bcl-X_L_ in the human breast cancer tissues and present evidences for the inhibitory effects of Bcl-X_L_, but not Bcl-2, on the TRAIL-induced apoptosis of tumour cell lines.

## MATERIALS AND METHODS

### Cell line and DNA transfection

MDA-MB-231, SK-BR-3, Jurkat, and Jurkat32H cells were grown in RPMI 1640 containing 10% fetal bovine serum (FBS). HeLa cells were incubated with Dulbecco's modified Eagle's Medium with 10% FBS. HeLa cells and Jurkat cells permanently expressing either Bcl-2 (HeLa/Bcl-2) or Bcl-X_L_ (HeLa/Bcl-X_L_) were established by transfection of expression plasmids of human Bcl-2 and Bcl-X_L_. Cells were transfected with Lipofect-AMINE PLUS™ reagent according to the recommended methods by the manufacturer (Gibco BRL, Grand Island, NY, USA) or with standard CaCl**_2_** methods. After 1 day, cells were grown in the presence of 700 *μ*g ml^−1^ G418 (Gibco BRL) or 1 *μ*g ml^−1^ puromycin (Sigma, St Louis, MO, USA) for 3 weeks. Each clone was examined for the expression of exogenous gene with Western blot analysis.

### Materials

Rhodamine 123, a cell-permeable mitotracker, was from Molecular Probe Inc. (Eugene, OR, USA). The fluorogenic caspase substrates DEVD-aminomethylcoumarine (AMC), IETD-AMC, and LEHD-AMC were from Enzyme System Products (Livermore, CA, USA). Anticaspase-3 (SC-7148) and anti-Bcl-X_L_ (M-125, SC-7195) antibodies were purchased from Santa Cruz (Santa Cruz, CA, USA); anti-Bcl-2 antibody was from DAKO (Copenhagen, Denmark); anti-caspase-8 and -9 antibodies were previously described (Kim *et al*, 2000); anti-*α*-tubulin antibody was from Sigma; anti-rabbit IgG-horseradish peroxidase (HRP), anti-mouse IgG-HRP, and anti-goat IgG-HRP antibodies were from Santa Cruz. All other molecular biology grade reagents were from Sigma or New England Biolabs (Hertfordshire, England).

### Collection of surgical samples

Tissues were obtained from seven patients who were operated upon infiltrating ductal carcinoma of breast at Department of Surgery, Seoul National University Hospital, Seoul, Korea. Fresh specimens of cancer core tissues and adjacent normal breast tissues from the same patient were immediately frozen in liquid nitrogen and stored at −80°C for Western blot analysis. For the immunohistochemical analysis, samples were formalin-fixed and paraffin-embedded. Parallel samples were processed for histologic examination.

### TNF-related apoptosis-inducing ligand preparation

TNF-related apoptosis-inducing ligand preparation was previously described by Cha *et al* (1999). Briefly, truncated human TRAIL (amino acid 114–281) in pET-3a plasmid was expressed in BL21 (DE3) by 1 mM isopropyl-beta-D-thiogalactoside. After sonication, TRAIL were isolated as insoluble aggregates by centrifugation and solubilised in a buffer containing 20 mM sodium phosphate (pH 7.6), 6 M guanidine-HCL, and 1 mM dithiothreitol (DTT). The denatured proteins were refolded by a rapid 10-fold dilution with a buffer solution containing 20 mM sodium phosphate (pH 7.6) and 1 mM DTT, followed by overnight dialysis in the same buffer at 4°C. After removing aggregates, the supernatant solution was loaded on a SP Sepharose Fast Flow column (Amersham-Pharmacia, Piscataway, NJ, USA). Fractions eluted at 0.8–1.0 M NaCl gradient contained TRAIL almost exclusively as judged by SDS–PAGE.

### Cell-based functional screening to isolate inhibitors of TRAIL-induced apoptosis

#### (a) Retroviral infection of cDNA library and selection for TRAIL-resistant clones

A retroviral library containing 2 × 10^7^ independent cDNA inserts was constructed from RNA of human lymph node, thymus, spleen, and bone marrow by standard methods, using a retrovirus vector pTRA. Amphotropic retroviral packaging cells, φ NX-ampho, were transfected with 10 *μ*g of cDNA library using CaCl_2_ method and produced up to 2 × 10^6^ infectious units ml^−1^. Forty-eight hours after transfection, the supernatant was collected and used to infect Jurkat32H cells. In total, 10^9^ cells were infected for 48 h and treated with 50 ng ml^−1^ TRAIL. The resulting TRAIL-resistant clones were separated by single-cell sorter (MolFlow, Fort Collins, CO, USA). After amplification, cells were exposed to TRAIL for secondary screening and analysed with FACScalibur™ (Beckton Dickinson, Franklin lakes, NJ, USA).

#### (b) Rescue analysis of cDNA

Total RNA was isolated from the putative positive clones. Candidate cDNAs were then amplified with SuperScript™ One-step RT–PCR system (Gibco BRL) using the library-specific primers. The resulting PCR products were purified from agarose gels and subjected to DNA sequencing analysis.

### Apoptosis assay

Flow cytometry analysis was performed with fluorescence-activated cell sorter (FACS) after staining cells with 50 *μ*g ml^−1^ pro-pidium iodide. Cell viability was also determined by 0.04% trypan blue exclusion assay or MTT assay. Viability of the transfectant was assessed as follows: cells grown on cover glasses were trans-fected with both pEGFP and effector expression plasmid for 1 day and then incubated with TRAIL for the indicated times. Cell viability was then determined based on the morphology of GFP-positive cells under a fluorescence microscope (Zeiss, Jena, Germany).

### Western blot analysis

In total, 30–50 *μ*g of cell extracts was subjected to SDS–PAGE in a buffer containing 60 mM Tris-Cl (pH 6.8), 1% SDS, 10% glycerol, and 0.5% *β*-mercaptoethanol, and then transferred to PVDF membranes using Semi-Dry Transfer system (Bio-Rad). The membranes were blocked with TBST buffer (20 mM Tris-Cl pH 7.5, 150 mM NaCl, 0.2% Tween-20) containing 5% nonfat dried milk, incubated for 2 h with the primary antibodies, and for an additional 2 h with HRP-conjugated secondary antibodies. Proteins were then visualised using Enhanced Chemiluminescence (ECL™, Amersham-Pharmacia).

### Immunohistochemical analysis

Immunohistochemical staining was performed by the ABC method using formalin-fixed, paraffin-embedded tissue sections. Five micrometer thick tissue sections mounted on silanised slides were deparaffinised in xylene followed by sequential washes in graded ethanol to phosphate-buffered saline (PBS). The samples were pretreated for 15 min in 10 mM sodium citrate, pH 7.0 and endogenous peroxidase activity was blocked with 3% H_2_O_2_ for 15 min. The slides were incubated with the primary antibodies and then with a biotinylated link antibody (DAKO) for 30 min followed by incubation in an avidin/biotinylated HRP solution. The samples were exposed to diaminobenzidine for 6 min, counterstained with Mayer's haematoxylin, and mounted in Permount (Fisher Scientific, NJ, USA).

### Caspase activity assays

Cells (1 × 10^7^) were resuspended in isolation buffer (20 mM HEPES–KOH, pH 7.6, 100 mM KCl, 0.5 mM Na-EDTA, 2 mM *β*-mercaptoethanol, 0.1 mM PMSF, 10 *μ*g ml^−1^ leupeptin, 25 *μ*g ml^−1^ ALLN). After incubation for 10 min at 4°C, the cells were disrupted by 20–30 strokes with a homogeniser and clarified by centrifugation for 1 h at 100 000 **g**. Enzymatic reactions were carried out at 37°C in reaction buffer (0.1 M HEPES, 2 mM DTT, 0.1% Chaps, 1% sucrose) containing 20 *μ*g protein and either 50 *μ*M DEVD-AMC or IETD-AMC. AMC fluorescence (480 nm emission excited by illumination at 360 nm) was measured using a fluorescence microplate reader (FL-600) (Bio-TEK instrument Inc., Winooski, VT, USA).

### Reverse transcription–polymerase chain reaction (RT)–PCR

Total RNA was extracted from the cells using MRC Trizol reagent (Cincinnati, OH, USA). cDNA was prepared from 1 *μ*g of total RNA using oligo(dT) primer and Moloney murine leukaemia virus reverse transcriptase (Gibco BRL), and amplified by PCR with Taq DNA polymerase. Primers used were: DR4, 5′-ctgcaggtcgtacctagctcagctgcaaccatc-3′ and 5′-cgtgaggtccagctgcctcatgagctggtcc-3′; DR5, 5′-caggactatagcactcactggaatgacctcc-3′ and 5′-cctcaatcttctgcttggcaagtctctctcc-3′; TRAIL, 5′-agcctgggacagacctgcgtgctgatcgtg-3′ and 5′-aactggcttcatggtccatgtctatcaagt-3′; *β*-actin, 5′-gagggaaatcgtgcgtgacat-3′ and 5′-acatctgctggaaggtggaca-3′.

## RESULTS

### Isolation of Bcl-X_L_ as an inhibitor of TRAIL-induced apoptosis by functional screening

**Figure 1:**
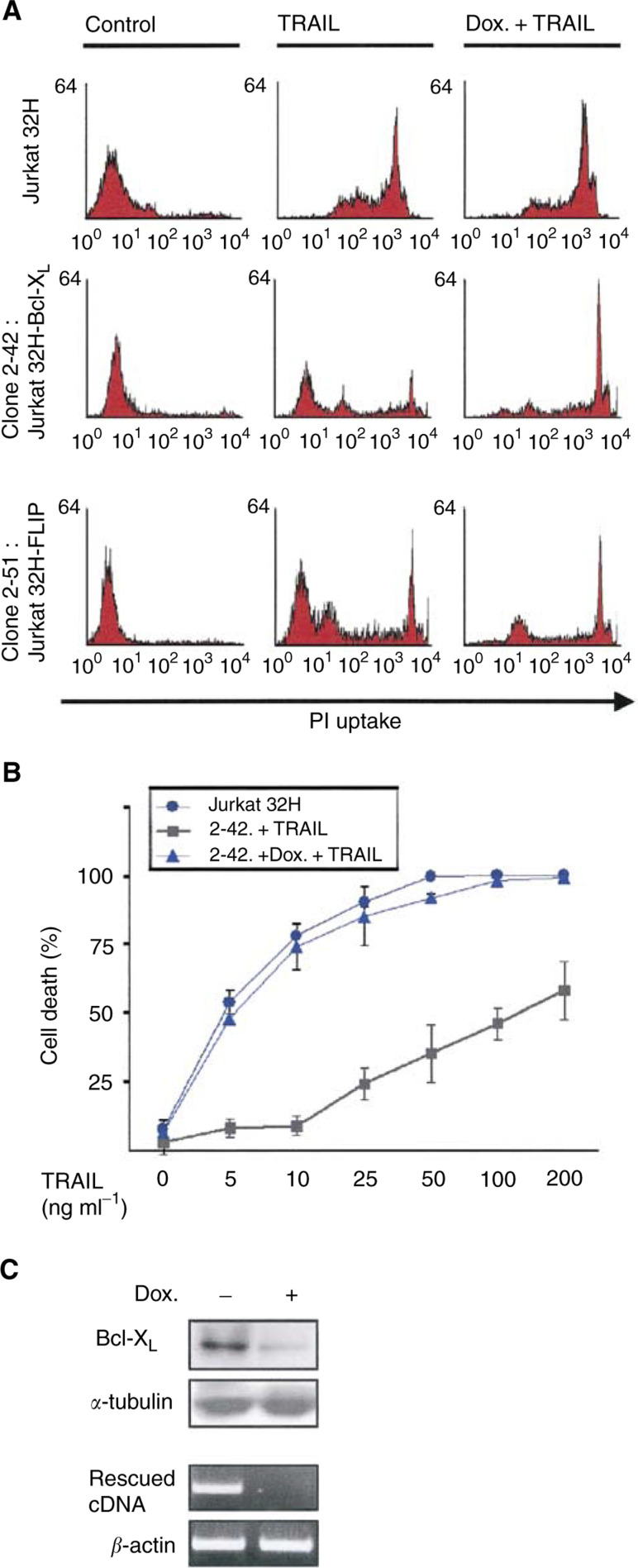
Doxycycline-dependent sensitivity of Jurkat32H cells harbouring Bcl-X_L_ to TRAIL-triggered apoptosis. (**A**) By cell-based functional screening of cDNA library, Jurkat32H ‘clone 2-42’ harbouring Bcl-X_L_ and ‘clone 2-51’ harbouring FLIP were selected and isolated as showing resistance to TRAIL. Each clone was left untreated (control) or exposed to TRAIL (50 ng ml^−1^) in the presence or absence of doxycycline (Dox, 50 ng ml^−1^) for 48 h. Cells were then incubated with 50 *μ*g ml^−1^ propidium iodide and analysed by FACS. (**B**) Jurkat32H ‘clone 2-42’ was incubated for 48 h with increasing concentrations of TRAIL in the presence or absence of doxycycline and cell death was then evaluated by trypan blue exclusion. (**C**) Western blot (Bcl-X_L_) and RT–PCR (rescued cDNA) analyses of Jurkat32H ‘clone 2-42’ after incubation with doxycycline for 48 h.

To isolate genes conferring resistance to TRAIL-induced apoptosis, we have screened cDNA libraries by functional genomics. Jurkat32H cells, which were modified for tetracycline off-inducible expression system, were infected with a retroviral human cDNA library and exposed to TRAIL. We used 50 ng ml^−1^ of TRAIL to induce 100% of cell death after 48 h (Figure 1B

). We achieved 30–40% infection as determined by doping of the library with marker retroviruses pTRA-GFP. TNF-related apoptosis-inducing ligand-resistant clones were selected out of 10^9^ cells by exposing to TRAIL for 48 h and isolated by FACS. DNA sequencing analysis of the cDNAs rescued from 11 TRAIL-resistant clones revealed that two of the TRAIL-resistant clones expressed Bcl-X_L_ and eight clones expressed FLICE-inhibitory protein (FLIP). Two of such clones, Jurkat32H ‘clone 2-42’ (Jurkat32H-Bcl-X_L_) and Jurkat32H ‘clone 2-51’ (Jurkat32H-FLIP), were analysed by flow cytometry for the resistance to TRAIL (Figure 1A). Expression of the exogenous gene in the inducible expression plasmid significantly protected Jurkat cells from apoptosis, showing comparable propidium iodide uptake to the control, while treatment with doxycycline sensitised the cells to TRAIL. Quantitative determination of apoptosis showed that expression of exogenous Bcl-X_L_ suppressed various concentrations of TRAIL-induced apoptosis up to 200 ng ml^−1^ (Figure 1B). Doxycycline-dependent expression pattern of Bcl-X_L_ in Jurkat32H-Bcl-X_L_ cells was confirmed by Western blotting and RT–PCR analysis (Figure 1C). These results indicate that the increased expression of Bcl-X_L_ confers resistance to TRAIL.

### Increased expression of Bcl-X_L_ in human breast cancer tissues

**Figure 2:**
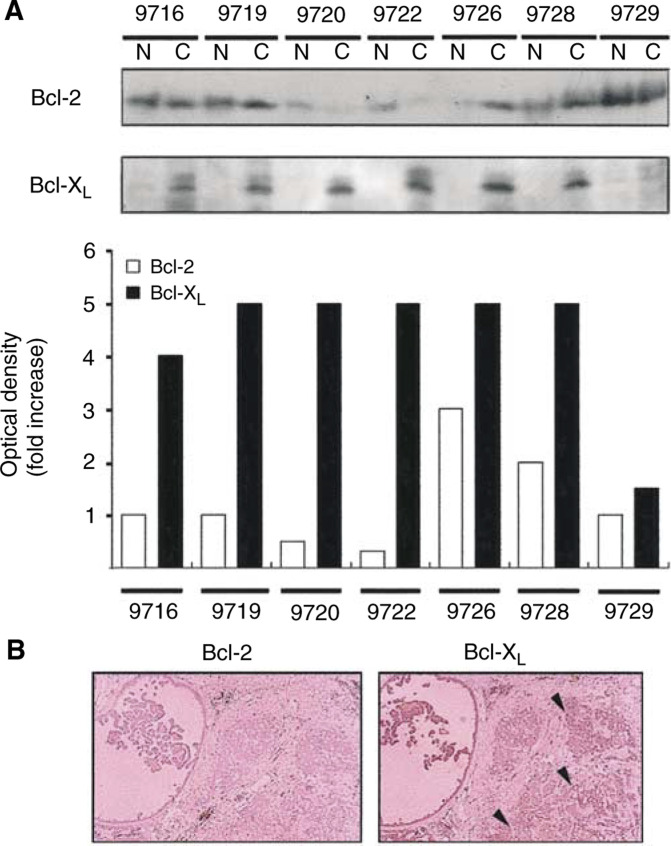
Upregulation of Bcl-X_L_ in human breast cancers. (**A**) Western blot analysis showing expression levels of Bcl-X_L_ and Bcl-2 in the extracts of normal (N) and breast cancer tissues (C). Numbers indicate individual human breast cancer patient from which tissues were derived. Relative level of Bcl-2 (open box) and Bcl-X_L_ (closed box) proteins were determined with densitometry using BIO-RAD ‘Quantity One’ image software. (**B**) Immunohistochemical analysis showing the expression pattern of Bcl-2 and Bcl-X_L_ in the tissue sections prepared from human breast cancer patients. Arrowheads indicate densely stained area with anti-Bcl-X_L_ antibody.

Based on the screening results, we examined the expression levels of Bcl-X_L_ and Bcl-2 in tissue extracts prepared from the human breast cancer patients (Figure 2

). Western blot analysis showed that in contrast to Bcl-2, expression of Bcl-X_L_ was markedly increased in breast cancer tissues compared to normal; six out of seven patients showed upregulated expression pattern of Bcl-X_L_ (Figure 2A). Densitometric analysis indicated that Bcl-X_L_ was increased 4–5-fold in cancer tissues. Similarly, a significant difference in the expression patterns of Bcl-X_L_ and Bcl-2 was observed by immunostaining of the tumour samples using anti-Bcl-X_L_ and anti-Bcl-2 antibodies (Figure 2B). Tumour samples from patients with locally advanced breast cancer were more specifically stained by anti-Bcl-X_L_ antibody than anti-Bcl-2 antibody, consistent with the result of Western blot analysis. Note the low basal immunoreactive signal of Bcl-2 compared to considerable level of Bcl-X_L_ in the tumour tissues. These results indicate that Bcl-X_L_ is highly upregulated in the breast cancer tissues.

### Different sensitivity of MDA-MB-231 and SK-BR-3, human breast tumour cell lines, to TRAIL

**Figure 3:**
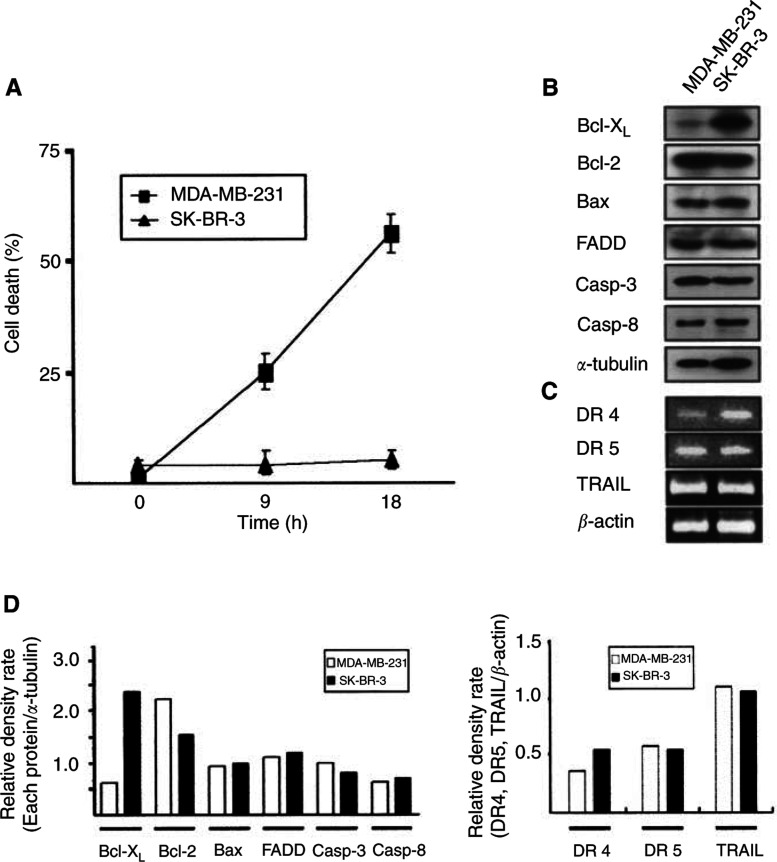
Different sensitivity of MDA-MB-231 and SK-BR-3 human breast tumour cell lines to TRAIL. (**A**) After exposure to 80 ng ml^−1^ TRAIL for the indicated times, cell viability was determined by trypan blue exclusion assay; values represent means ±s.d. of three independent experiments. (**B**) Western blot analysis showing expression levels of Bcl-X_L_, Bcl-2, Bax, FADD, and caspases in MDA-MB-231 and SK-BR-3 cells. (**C**) RT–PCR analysis showing expression level of DR4, DR5, and TRAIL. Total RNA was isolated and analysed by RT–PCR using gene-specific primers. (**D**) Densitometric analysis. The relative ratio of the signals detected by Western blot in (**B**) and RT–PCR analysis in (**C**) was determined using *α*-tubulin (*left panel*) and *β*-actin (*right panel*) as controls, respectively.

To explore the effects of increased expression of Bcl-X_L_ on TRAIL-induced responses, several human breast tumour cell lines were examined for the expression levels of Bcl-2 and Bcl-X_L_, and their sensitivities to TRAIL (data not shown). Among them, MDA-MB-231 and SK-BR-3 cell lines were selected by their different expression levels of Bcl-X_L_ and sensitivities to TRAIL. The death rates of MDA-MB-231 cells exposed to TRAIL were 24 and 55% at 9 and 18 h, respectively (Figure 3A

). On the contrary, SK-BR-3 cells were resistant to TRAIL. Examination of expression level with Western blotting and RT–PCR followed by densitometric analysis showed that Bcl-X_L_ was upregulated 3.8-fold in SK-BR-3 cells compared with MDA-MB-231 cells, while Bcl-2, Bax, FADD, caspase-3, caspase-8, DR4, and DR5 were not significantly different (Figure 3B–D). TRAIL was expressed in those cell lines without any detectable differences (Figure 3D, *right panel*).

**Figure 4:**
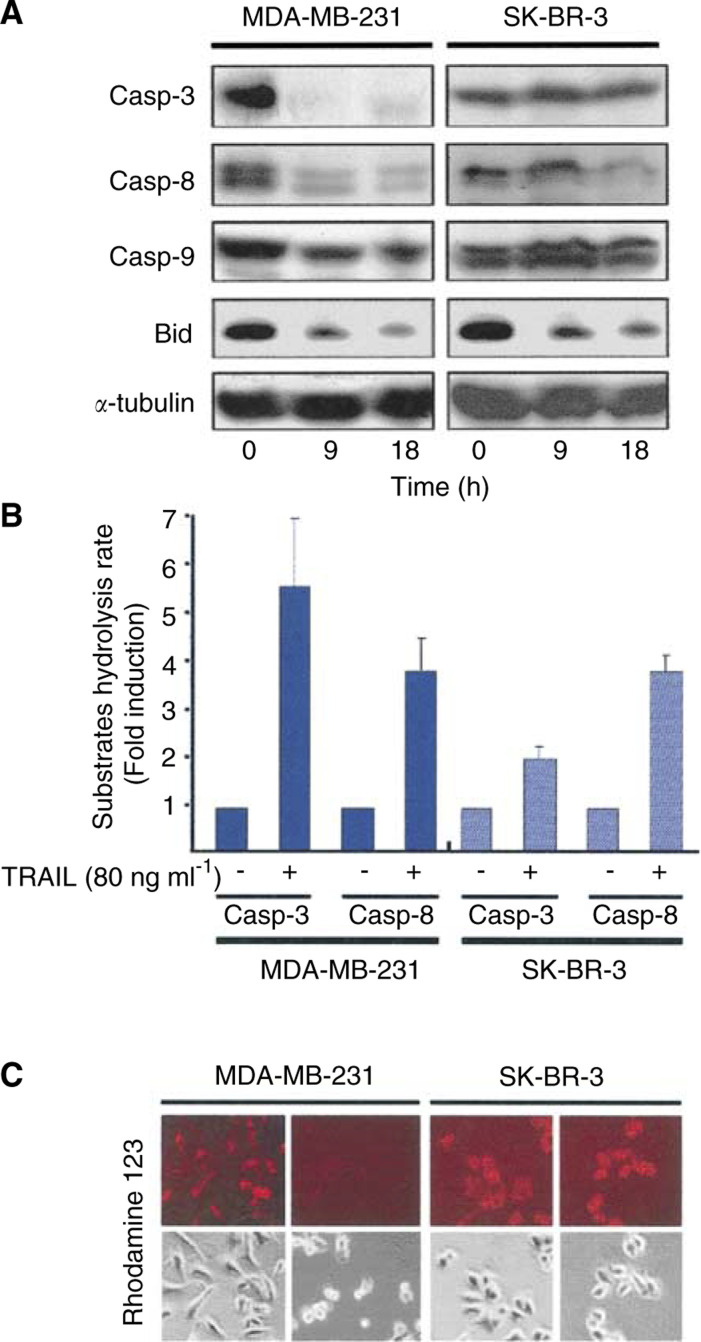
Caspase activation and dissipation of mitochondrial membrane potential in MDA-MB-231 and SK-BR-3 cells. (**A**) Western blot analysis showing proteolytic activation of caspases and cleavage of Bid. Cell lysates were prepared from cells exposed to TRAIL for the indicated times, separated by SDS–PAGE, and analysed by immunoblotting using the indicated antibodies including caspase (Casp). (**B**) Caspase activity assay. Cells (1 × 10^7^ cells) were left untreated or treated for 18 h with 80 ng ml^−1^ TRAIL. Cell extracts were prepared as described in Materials and Methods and assayed for caspase activity using the fluorogenic substrates DEVD-AMC (caspase-3-like protease) and IETD-AMC (caspase-8-like protease). Caspase activities of control cells were adjusted to arbitrary unit 1.0 and relative caspase activities (fold induction) were represented at left. (**C**) Lack of mitochondrial membrane potential dissipation in SK-BR-3 cells exposed to TRAIL. MDA-MB-231 and SK-BR-3 cells were left untreated or exposed to TRAIL for 18 h and mitochondrial membrane potentials were then measured using Rhodamine 123.

We have then examined activation of caspase in TRAIL-resistant SK-BR-3 cells and compared it to that of TRAIL-sensitive MDA-MB-231 cells. Western blot analysis revealed that proforms of caspase-3 and -8 disappeared within 9 h of exposure to TRAIL in MDA-MB-231 cells, indicating that those caspases were proteolytically activated in the TRAIL-sensitive cells (Figure 4A

). Caspase-9 appeared to be activated but less effectively. Indeed, processed and active forms of caspases, including large subunit (p20) and small subunit (p18) were detected at every time point following exposure to TRAIL (data not shown). Interestingly, procaspase-8, but not caspase-3 and -9, in the TRAIL-resistant SK-BR-3 cells was apparently reduced like MDA-MB-231 cells. Bid, a substrate of caspase-8, also decreased in both cell lines exposed to TRAIL. Determination of enzymatic activation using fluorogenic substrate showed that TRAIL treatment induced activation of caspase-8 about 3–4-fold in both cell lines (Figure 4B). Caspase-3 was effectively activated in MDA-MB-231 cells with 5.5-fold, consistent with the result of Western blot analysis (Figure 4A), while only marginal activation of caspase-3 was observed in SK-BR-3 cells. These results led us to propose that caspase-8 was equally activated in both cell lines, whereas caspase-3 activation was suppressed in TRAIL-resistant SK-BR-3 cells.

Since Bcl-X_L_ is known to suppress mitochondria-mediated cell death, mitochondrial membrane potential was examined with mitotracker Rhodamine 123 after exposure to TRAIL (Figure 4C). Dissipation of mitochondrial membrane potential was observed in TRAIL-sensitive MDA-MB-231 cells, but not in TRAIL-resistant SK-BR-3 cells, indicating that mitochondria-mediated death event is defective in SK-BR-3 cells expressing high level of Bcl-X_L_.

### Attenuation of TRAIL-induced apoptosis by overexpression of Bcl-X_L_ in TRAIL-sensitive MDA-MB-231 cells

**Figure 5:**
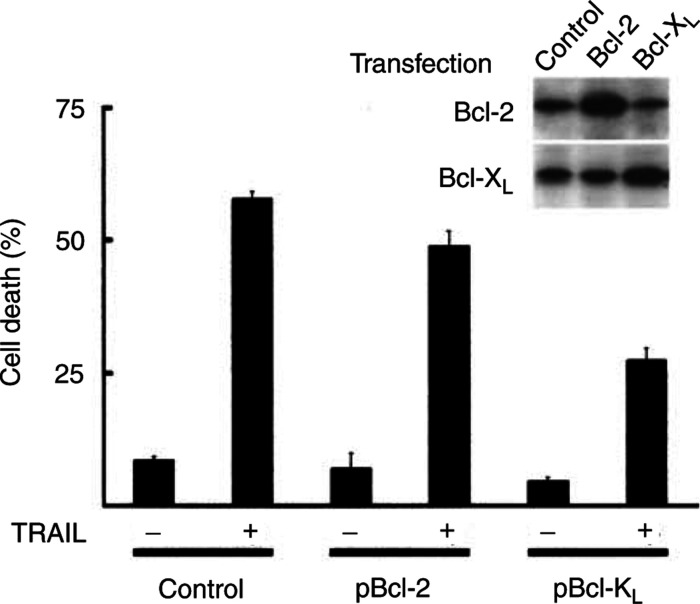
Ectopic expression of Bcl-X_L_, but not Bcl-2, rescued MDA-MB-231 cells from TRAIL-induced apoptosis. MDA-MB-231 cells were transiently transfected with pEGFP and either pcDNA3 (control), pBcl-2, or pBcl-X_L_. pEGFP was included in every transfection reactions with a ratio of 1 : 3. After 24 h, cells were incubated with TRAIL for 18 h and death rates were determined based on the morphology of GFP-positive cells under a fluorescence microscope. Cell extracts prepared from each transfectant were subjected to Western blot analysis.

**Figure 6:**
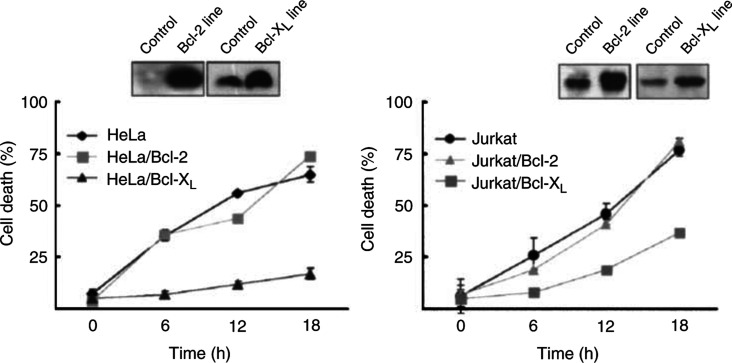
Differential effect of Bcl-X_L_ and Bcl-2 on TRAIL-induced apoptosis. HeLa and Jurkat cells permanently expressing Bcl-X_L_ or Bcl-2 were exposed to TRAIL (80 ng ml^−1^) for the indicated times and cell viability was determined by trypan blue and MTT assays. Expression levels of Bcl-X_L_ or Bcl-2 in the respective cell line were examined by Western blot analysis.

We have then addressed whether increased expression of Bcl-X_L_ in TRAIL-sensitive MDA-MB-231 cells suppressed TRAIL-induced apoptosis. MDA-MB-231 cells were transiently transfected with Bcl-2 or Bcl-X_L_ expression vectors and subsequently exposed to TRAIL (Figure 5

). Determination of cell viability showed that MDA-MB-231 cells expressing Bcl-X_L_ became resistant to TRAIL; death rates decreased from 58 to 29%. Interestingly, Bcl-2 was less potent to suppress TRAIL-induced apoptosis of MDA-MB-231 cells (death rates, 58 to 49%) compared with Bcl-X_L_. Similar inhibitory effects of Bcl-X_L_ and Bcl-2 were further observed in HeLa, human cervical tumour cells, and Jurkat, human lymphoma cells, permanently overexpressing Bcl-X_L_ or Bcl-2 (Figure 6

). Expression levels of Bcl-2 or Bcl-X_L_ in these stable cell lines were 3–4-fold higher than control cells, similar ratio with the increased expression of Bcl-X_L_ in the breast cancer tissues as shown in Figure 2. HeLa and Jurkat cells were sensitive to TRAIL, exhibiting 60–70% of death rates when exposed to TRAIL. Forced expression of Bcl-2 in those stable cells did not suppress the death rates. On the contrary, expression of Bcl-X_L_ in HeLa or Jurkat cells substantially reduced TRAIL-induced apoptosis, indicating differential inhibitory activities of Bcl-X_L_ and Bcl-2 in TRAIL-mediated cell death.

## DISCUSSION

In the present study, we have screened and characterised genes inhibiting TRAIL-induced apoptosis and isolated several genes including FLIP and antiapoptotic Bcl-2 family proteins, including Bcl-X_L_ and Mcl-1, but not Bcl-2. Unlike Bcl-2, Bcl-X_L_ is highly upregulated in human breast cancer tissues and effective to suppress TRAIL-triggered apoptosis in several tumour cell lines. Thus, the increased expression of Bcl-X_L_ in the breast tumours and cell lines may desensitise the cells to TRAIL.

Others and we have previously shown that caspase-8 and FADD played a critical role in TRAIL-induced apoptosis (Bodmer *et al*, 2000; Kim *et al*, 2000; Kischkel *et al*, 2000). Also FLIP, a caspase-8 interacting inhibitory protein, was recently reported to suppress TRAIL-induced apoptosis (Harper *et al*, 2001), consistent with our screening results. However, there were no detectable differences in the expression levels of caspase-8 and FADD between SK-BR-3 and MDA-MB-231 breast tumour cells showing different sensitivities to TRAIL. In addition, TRAIL-mediated activation of caspase-8 and Bid cleavage in TRAIL-resistant SK-BR-3 cells indicates that TRAIL receptor and its associated adaptor molecules such as FLIP linking to caspase-8 appeared to be functionally normal. Instead, Bcl-X_L_ in SK-BR-3 cells is likely to be one of the components in the signalling complexes contributing to the resistance to TRAIL, probably by interfering with mitochondria-mediated apoptotic pathway, though we could not examine the sensitivity of the primary culture cells directly derived from the breast cancer tissues to TRAIL.

Though the biochemical mechanisms by which members of the Bcl-2 family of proteins inhibit apoptosis remain enigmatic, the following properties have been proposed to play a role in the modulation of apoptosis (Reed, 1997; Hengartner, 2000). First, the ability of some members to form pore or channel, through which cytochrome c and other intermembrane proteins escape with low selective permeability, similar to some pore-forming bacterial toxins (Minn *et al*, 1997). Second, the ability of the different members of this family to function as docking proteins able to bind each other to form homo- or heterodimers as well as bind to other proteins (Sato *et al*, 1994; Sedlak *et al*, 1995). Thus, the ratio between proapoptotic and antiapoptotic molecules in a particular cell may then determine the response to an apoptotic stimulus. However, there was no detectable difference in the expression level of Bax, a proapoptotic molecule interacting with Bcl-2 and Bcl-X_L_, between cell lines used in this study.

Interestingly, Bcl-2 did not show comparable antiapoptotic activity on TRAIL-mediated apoptosis, indicating that the death signalling activated by TRAIL was not susceptible to the inhibition mediated by Bcl-2 in the tumour cells we have examined. However, the ability of Bcl-2 to suppress apoptosis triggered by TRAIL is controversial. Human glioma and prostate cancer cells lost their sensitivity to TRAIL by overexpressing Bcl-2 (Rieger *et al*, 1998; Munshi *et al*, 2001; Rokhlin *et al*, 2001). In contrast, other groups have shown that Bcl-2-transfected 8226 and ARP-1 myeloma cells still remained sensitive to TRAIL (Gazitt, 1999a,1999b) and that Bcl-2 failed to block cytochrome *c* release after exposure to TRAIL (Keogh *et al*, 2000; Walczak *et al*, 2000). Although the discrepancy between those observations is not clearly resolved, it might be arisen from difference in cell types or in the relative expression level of Bcl-2 family proteins including Bcl-2 itself.

Although function of Bcl-2 family proteins looks similar, increasing numbers of evidences have suggested that prosurvival activities of Bcl-2 and Bcl-X_L_ are differently regulated. Bcl-2 and Bcl-X_L_ may function at distinct sites (Chinnaiyan *et al*, 1997; El-Assaad *et al*, 1998). It is believed that death receptor-induced apoptotic pathway generally bypasses the Bcl-2-inhibitable steps, whereas Bcl-2 protects against diverse cytotoxic insults, for example, *γ*- and ultraviolet-irradiation, cytokine withdrawal, dexamethasone, and cytotoxic drugs (Cory, 1995; Strasser *et al*, 1995; Yang and Korsmeyer, 1996; Chao and Korsmeyer, 1998). In contrast, Bcl-X_L_ is thought to be more potent to suppress death receptor-induced cell death pathway (Fernandez *et al*, 2000). Thus, cancer cells may increase expression of Bcl-X_L_ rather than Bcl-2 to be resistant to ligand-mediated cytotoxic stimuli including TRAIL. Taken together, we propose here that the increased expression of Bcl-X_L_ observed in the human breast tumours desensitises tumourigenic cells to apoptosis triggered by TRAIL.

## References

[bib1] Ashkenazi A, Pai RC, Fong S, Leung S, Lawrence DA, Marsters SA, Blackie C, Chang L, McMurtrey AE, Hebert A, DeForge L, Koumenis IL, Lewis D, Harris L, Bussiere J, Koeppen H, Shahrokh Z, Schwall RH (1999) Safety and antitumor activity of recombinant soluble Apo2 ligand. J Clin Invest 104: 155–162 10411544 10.1172/JCI6926 PMC408479

[bib2] Bodmer JL, Holler N, Reynard S, Vinciguerra P, Schneider P, Juo P, Blenis J, Tschopp J (2000) TRAIL receptor-2 signals apoptosis through FADD, caspase-8. Nat Cell Biol 2: 241–243 10783243 10.1038/35008667

[bib3] Cha SS, Shin HC, Choi KY, Oh BH (1999) Expression, purification, and crystallization of recombinant human TRAIL. Acta Crystallogr D Biol Crystallogr 55: 1101–1104 10216319 10.1107/s090744499900164x

[bib4] Chao DT, Korsmeyer SJ (1998) BCL-2 family: regulators of cell death. Annu Rev Immunol 16: 395–419 9597135 10.1146/annurev.immunol.16.1.395

[bib5] Chinnaiyan AM, O'Rourke K, Lane BR, Dixit VM (1997) Interaction of CED-4 with CED-3 and CED-9: a molecular framework for cell death. Science 275: 1122–1126 9027312 10.1126/science.275.5303.1122

[bib6] Cory S (1995) Regulation of lymphocyte survival by the bcl-2 gene family. Annu Rev Immunol 13: 513–543 7612233 10.1146/annurev.iy.13.040195.002501

[bib7] El-Assaad W, El-Sabban M, Awaraji C, Abboushi N, Dbaibo GS (1998) Distinct sites of action of Bcl-2 and Bcl-xL in the ceramide pathway of apoptosis. Biochem J 336: 735–741 9841888 10.1042/bj3360735 PMC1219927

[bib8] Fernandez Y, Espana L, Manas S, Fabra A, Sierra A (2000) Bcl-xL promotes metastasis of breast cancer cells by induction of cytokines resistance. Cell Death Differ 7: 350–359 10773819 10.1038/sj.cdd.4400662

[bib9] Gazitt Y (1999a) TRAIL is a potent inducer of apoptosis in myeloma cells derived from multiple myeloma patients and is not cytotoxic to hematopoietic stem cells. Leukemia 13: 1817–1824 10557057 10.1038/sj.leu.2401501

[bib10] Gazitt Y, Shaughnessy P, Montgomery W (1999b) Apoptosis-induced by TRAIL and TNF-alpha in human multiple myeloma cells is not blocked by Bcl-2. Cytokine 11: 1010–1019 10623426 10.1006/cyto.1999.0536

[bib11] Green DR, Reed JC (1998) Mitochondria and apoptosis. Science 281: 1309–1312 9721092 10.1126/science.281.5381.1309

[bib12] Harper N, Farrow SN, Kaptein A, Cohen GM, MacFarlane M (2001) Modulation of tumor necrosis factor apoptosis-inducing ligand-induced NF-kappa B activation by inhibition of apical caspases. J Biol Chem 276: 34 743–34 752 10.1074/jbc.M105693200 11461927

[bib13] Harris MH, Thompson CB (2000) The role of the Bcl-2 family in the regulation of outer mitochondrial membrane permeability. Cell Death Differ 7: 1182–1191 11175255 10.1038/sj.cdd.4400781

[bib14] Hengartner M (2000) The biochemistry of apoptosis. Nature 407: 770–777 11048727 10.1038/35037710

[bib15] Jo M, Kim TH, Seol DW, Esplen JE, Dorko K, Billiar TR, Strom SC (2000) Apoptosis induced in normal human hepatocytes by tumor necrosis factor-related apoptosis-inducing ligand. Nat Med 6: 564–567 10802713 10.1038/75045

[bib16] Keogh SA, Walczak H, Bouchier-Hayes L, Martin SJ (2000) Failure of Bcl-2 to block cytochrome *c* redistribution during TRAIL-induced apoptosis. FEBS Lett 471: 93–98 10760520 10.1016/s0014-5793(00)01375-2

[bib17] Kim IK, Chung CW, Woo HN, Hong GS, Nagata S, Jung YK (2000) Reconstitution of caspase-8 sensitizes JB6 cells to TRAIL. Biochem Biophys Res Commun 277: 311–316 11032723 10.1006/bbrc.2000.3673

[bib18] Kischkel FC, Lawrence DA, Chuntharapai A, Schow P, Kim KJ, Ashkenazi A (2000) Apo2L/TRAIL-dependent recruitment of endogenous FADD and caspase-8 to death receptors 4 and 5. Immunity 12: 611–620 10894161 10.1016/s1074-7613(00)80212-5

[bib19] Lawrence D, Shahrokh Z, Marsters S, Achilles K, Shih D, Mounho B, Hillan K, Totpal K, DeForge L, Schow P, Hooley J, Sherwood S, Pai R, Leung S, Khan L, Gliniak B, Bussiere J, Smith CA, Strom SS, Kelley S, Fox JA, Thomas D, Ashkenazi A (2001) Differential hepatocyte toxicity of recombinant Apo2L/TRAIL versions. Nat Med 7: 383–385 11283636 10.1038/86397

[bib20] Leverkus M, Neumann M, Mengling T, Rauch CT, Brocker EB, Krammer PH, Walczak H (2000) Regulation of tumor necrosis factor-related apoptosis-inducing ligand sensitivity in primary and transformed human keratinocytes. Cancer Res 60: 553–559 10676636

[bib21] Minn AJ, Vélez P, Schendel SL, Liang H, Muchmore SW, Fesik SW, Fill M, Thompson CB (1997) Bcl-x(L) forms an ion channel in synthetic lipid membranes. Nature 385: 353–357 9002522 10.1038/385353a0

[bib22] Mongkolsapaya J, Cowper AE, Xu XN, Morris G, McMicahel AJ, Bell JI, Screaton GR (1998) Lymphocyte inhibitor of TRAIL (TNF-related apoptosis-inducing ligand): a new receptor protecting lymphocytes from the death ligand TRAIL. J Immunol 160: 3–6 9551946

[bib23] Munshi A, Pappas G, Honda T, McDonnell TJ, Younes A, Li Y, Meyn RE (2001) TRAIL (APO2-L) induces apoptosis in human prostate cancer cells that is inhibitable by Bcl-2. Oncogene 20: 3757–3765 11439339 10.1038/sj.onc.1204504

[bib25] Pan G, Ni J, Wei YF, Yu G, Gentz R, Dixit VM (1997a) An antagonist decoy receptor and a death domain-containing receptor for TRAIL. Science 277: 815–818 9242610 10.1126/science.277.5327.815

[bib26] Pan G, O'Rouke K, Chinnaiyan AM, Gentz R, Ebner R, Ni J, Dixit VM (1997b) The receptor for the cytotoxic ligand TRAIL. Science 276: 111–113 9082980 10.1126/science.276.5309.111

[bib27] Pitti RM, Marsters SA, Ruppert A, Donahue CJ, Moore A, Ashkenazi A (1996) Induction of apoptosis by Apo-2 ligand, a new member of the tumor necrosis factor cytokine family. J Biol Chem 271: 12 687–12 690 10.1074/jbc.271.22.12687 8663110

[bib28] Qin J, Chaturvedi V, Bonish B, Nickoloff BJ (2001) Avoiding premature apoptosis of normal epidermal cells. Nat Med 7: 385–386 11283637 10.1038/86401

[bib29] Reed JC (1997) Double identity for proteins of the Bcl-2 family. Nature 387: 773–776 9194558 10.1038/42867

[bib30] Rieger J, Naumann U, Glaser T, Ashkenazi A, Weller M (1998) APO2 ligand: a novel lethal weapon against malignant glioma? FEBS Lett 427: 124–128 9613612 10.1016/s0014-5793(98)00409-8

[bib31] Rokhlin OW, Guseva N, Tagiyev A, Knudson CM and Cohen MB (2001) Bcl-2 oncoprotein protects the human prostatic carcinoma cell line PC3 from TRAIL-mediated apoptosis. Oncogene 20: 2836–2843 11420695 10.1038/sj.onc.1204410

[bib32] Sato T, Hanada M, Bodrug S, Irie S, Iwama N, Boise LH, Thompson CB, Golemis E, Fong L, Wang HG, Reed JC (1994) Interactions among members of the Bcl-2 protein family analyzed with a yeast two-hybrid system. Proc Natl Acad Sci USA 91: 9238–9242 7937747 10.1073/pnas.91.20.9238 PMC44787

[bib33] Sedlak TW, Oltvai ZN, Yang E, Wang K, Boise LH, Thompson CB, Korsmeyer SJ (1995) Multiple Bcl-2 family members demonstrate selective dimerizations with Bax. Proc Natl Acad Sci USA 92: 7834–7838 7644501 10.1073/pnas.92.17.7834 PMC41240

[bib34] Sheridan JP, Marsters SE, Pitti RM, Gurney A, Skubatch M, Baldwin D, Ramarkrishnan L, Gray CL, Baker K, Wood WI, Goddard AD, Godowski P, Ashkenazi A (1997) Control of TRAIL-induced apoptosis by a family of signalling and decoy receptors. Science 277: 818–821 9242611 10.1126/science.277.5327.818

[bib35] Strasser A, Harris AW, Huang DCS, Krammer PH, Cory S (1995) Bcl-2 and Fas/APO-1 regulate distinct pathways to lymphocyte apoptosis. EMBO J 14: 6136–6147 8557033 10.1002/j.1460-2075.1995.tb00304.x PMC394738

[bib36] Thornberry NA, Lazebnik Y (1998) Caspases: enemies within. Science 281: 1312–1316 9721091 10.1126/science.281.5381.1312

[bib37] Vander-Heiden MG, Chandel NS, Williamson EK, Schumacker PT, Thompson CB (1997) Bcl-xL regulates the membrane potential and volume homeostasis of mitochondria. Cell 91: 627–637 9393856 10.1016/s0092-8674(00)80450-x

[bib39] Walczak H, Bouchon A, Stahl H, Krammer PH (2000) Tumor necrosis factor-related apoptosis-inducing ligand retains its apoptosis-inducing capacity on Bcl-2- or Bcl-xL-overexpressing chemotherapy-resistant tumor cells. Cancer Res 60: 3051–3057 10850456

[bib38] Walczak H, Miller RE, Ariail K, Gliniak B, Griffith TS, Kubin M, Chin W, Jones J, Woodward A, Le T, Smith C, Smolak P, Goodwin RG, Rauch CT, Schuh JC, Lynch DH (1999) Tumoricidal activity of tumor necrosis factor-related apoptosis-inducing ligand *in vivo*. Nat Med 5: 157–163 9930862 10.1038/5517

[bib40] Wiley SR, Schooley K, Smolak PJ, Din WS, Huang CP, Nicholl JK, Sutherland GR, Smith TD, Rauch C, Smith CA, Goodwin RG (1995) Identification and characterization of a new member of the TNF family that induces apoptosis. Immunity 3: 673–682 8777713 10.1016/1074-7613(95)90057-8

[bib41] Wu GS, Burns TF, McDonald III ER, Jiang W, Meng R, Krantz ID, Kao G, Gan DD, Zhou JY, Muschel R, Hamilton SR, Spinner NB, Markowitz S, Wu G, El-Deiry WS (1997) KILLER/DR5 is a DNA damage-inducible p53-regulated death receptor gene. Nat Genet 17: 141–143 9326928 10.1038/ng1097-141

[bib42] Yang E, Korsmeyer SJ (1996) Molecular thanatopsis: a discourse on the BCL2 family and cell death. Blood 88: 386–401 8695785

